# Usefulness of Haemoglobin Level Combined with CAMI-STEMI Score for Predicting MACCE in Patients with Acute ST-Elevation Myocardial Infarction after PCI

**DOI:** 10.1155/2019/8534752

**Published:** 2019-07-22

**Authors:** Chengchun Tang, Erfei Luo, Dong Wang, Gaoliang Yan, Yong Qiao, Boqian Zhu, Bo Liu, Jiantong Hou

**Affiliations:** Department of Cardiology, Zhongda Hospital, Southeast University, Nanjing, 210009, China

## Abstract

Anaemia and high haemoglobin levels are common in ST elevation myocardial infarction (STEMI) patients, but the effect of the haemoglobin level on the prognosis of STEMI patients remains in dispute. This study aimed to evaluate the prognostic value of the haemoglobin level combined with the CAMI-STEMI score in STEMI patients after percutaneous coronary intervention (PCI). We included 360 STEMI patients who underwent PCI. The patients were divided into 3 groups according to the first haemoglobin value after PCI. Clinical characteristics and the incidence of major adverse cardiovascular and cerebral events (MACCE) during the follow-up period were recorded. The incidence of MACCE in the 3 groups increased with a decrease in the haemoglobin level. Multivariate regression analysis showed that the CAMI-STEMI score was an independent predictor of MACCE incidence at 30 days after PCI and that anaemia was an independent predictor of MACCE incidence at 6 months and 1 year after PCI. A high haemoglobin level was an independent predictor of MACCE incidence at 1 year after PCI. The area under receiver operating characteristic curves (AUCs) of the haemoglobin level, CAMI-STEMI score, and haemoglobin level combined with CAMI-STEMI score predicting the occurrence of MACCE in STEMI patients within 30 days after PCI were 0.604, 0.614, and 0.639, respectively. In conclusion, The CAMI-STEMI score was an independent predictor of MACCE incidence at 30 days after PCI. The haemoglobin level combined with the CAMI-STEMI score improved the predictive value of MACCE in STEMI patients within 30 days after PCI.* Trial Registration*. This trial was a prospective cohort study and registered with ChiCTR-ROC-17011542.

## 1. Background

Anaemia and high haemoglobin levels are common in ST elevation myocardial infarction (STEMI) patients, but the effect of the haemoglobin level on the prognosis of STEMI patients remains in dispute [1, 2]. Numerous observational studies have suggested that anaemia is an independent predictor of short-term cardiovascular events during hospitalization and within 30 days in patients with acute coronary syndrome (ACS) [3–5]. However, there were different views on the role of anaemia in forecasting the long-term prognosis of patients with ACS [6, 7]. The prognostic value of anaemia in patients with different types of myocardial infarction (STEMI and NSTEMI) was not the same [1]. Studies have shown that, in STEMI patients, anaemia should be considered as an additional risk factor at admission [5]. However, the correlation between elevated haemoglobin levels and cardiovascular risk remains unclear. Sabatine et al. reported that, in patients with ACS, both high and low haemoglobin levels were linked to increased rates of mortality [1]. In patients with ACS, the usefulness of risk score for predicting death and cardiovascular complications has been proved both in the short and long term [1, 8]. Early risk stratification was strongly recommended to guide therapeutic management and to improve outcome [9]. Yang et al. developed the China Acute Myocardial Infarction registry-ST Elevation Myocardial Infarction (CAMI-STEMI) score for the 2017 European Society of Cardiology (ESC). The CAMI-STEMI score can predict in-hospital mortality among Chinese STEMI patients, with similar performance to the well-established Thrombolysis in Myocardial Infarction (TIMI) score, while relying solely on simple and practical variables; that is, there is no need to ask the history or draw blood to check the biochemistry [10]. The purpose of this study was to explore the predictive value of the haemoglobin level combined with the CAMI-STEMI score on the prognosis of STEMI patients after PCI and to provide ideas for improving STEMI risk stratification and optimizing the treatment of myocardial infarction.

## 2. Methods

From June 2013 to April 2016, consecutive patients with STEMI admitted to Zhongda Hospital (Nanjing, People's Republic of China) and treated with PCI were enrolled. The inclusion criteria were as follows: patients with STEMI who were diagnosed based on the* guidelines for the diagnosis and treatment of acute ST segment elevation myocardial infarction in 2010 (China)* [11]. The exclusion criteria were as follows: a history of major surgery, trauma, and bleeding over the past three months; contraindications to anticoagulant and antiplatelet therapy; serious injury of liver and kidney; malignant tumour; and clinical data and coronary angiography being incomplete.

Anaemia was defined as a serum haemoglobin level of <120 g/L for men and <110 g/L for women [12]. The normal reference range of serum haemoglobin was defined as 120 g/L to 160 g/L for men and 110 g/L to 150 g/L for women [13]. The patients were divided into 3 groups according to the first haemoglobin value after PCI, group 1 (male: Hb<120 g/L, female: Hb<110 g/L; 42 cases), group 2 (male: 120 g/L ≤ Hb<160 g/L, female: 110 g/L≤Hb<150 g/L; 278 cases), and group 3 (male: Hb ≥160 g/L, female: Hb ≥150 g/L;40 cases). Haemoglobin was measured from venous blood 24 hours after PCI. Biochemical indices including blood sugar, blood lipid, and creatinine were measured.

The CAMI-STEMI score was composed of seven variables: female (1 point), heart rate (HR) ≥100 bmp (2 points), age ≥70 years (2 points), systolic blood pressure ≤115 mmHg (2 points), Killip class>1 (2 points), cardiac arrest (4 points), and anterior wall infarction (1 point) [10].

All patients were given aspirin (300 mg), ticagrelor (180 mg), or clopidogrel (300 mg) before operation, and aspirin (100 mg, QD), ticagrelor (90 mg, BID), or clopidogrel (75 mg, QD) were administered after surgery. Heparin was utilized routinely during surgery and as appropriate after surgery. Statins, beta blockers, nitrates, and angiotensin-converting enzyme inhibitors were routinely used in all three groups without contraindications.

The endpoints were the major adverse cardiac and cerebrovascular events (MACCE) during the follow-up period (30 days, 6 months, and 1 year after PCI). The MACCE included all-cause death, reconstruction of target vascular lesions, myocardial infarction during follow-up, unstable angina pectoris requiring hospitalization, heart failure, stroke, or transient cerebral ischaemia.

Each patient's baseline clinical data (including demographic data, previous medical history, and vital signs on admission), biochemical and angiographic variables, and ultrasonic cardiogram results were recorded. All patients were followed up by a cardiovascular physician and by telephone. All patients included in the study volunteered to participate in this clinical study and signed an informed consent form.

### 2.1. Statistical Analysis

Analyses were performed using SPSS software, version 16.0 (SPSS, Inc., Chicago, IL, USA). Continuous variables were expressed as the mean±SD or median (inter-quartile range). Categorical variables were expressed as frequencies with percentages. Survival was graphically represented using Kaplan-Meier curves. Differences in survival rates were compared using the log-rank test. Univariate and multivariate Cox proportional hazards models were used to identify study endpoint predictors. Variables with univariate P values <0.10 were selected for multivariate analysis and expressed as hazard ratio (HR) with 95% confidence intervals (CIs). Multivariate Cox regression analysis was performed using a forward stepwise method. The area under receiver operating characteristic curve (AUC) was used to indicate the predictive value of the target factor on individual prognosis. All tests were 2-tailed, and statistical significance was defined as P <0.05.

## 3. Results

This study selected 378 patients. Eighteen cases were lost to follow-up, for a lost-to-follow-up rate of 4.8%; thus, 360 patients were finally enrolled. Baseline clinical data are presented in Table 1. Biochemical and angiographic variables, and ultrasonic cardiogram results are shown in Table 2. There were statistically significant differences (P<0.05) among the three groups in terms of age, the proportion of female patients, body mass index (BMI), smoking history, the proportion of Killip class>1, blood potassium concentration, albumin, alanine aminotransferase (ALT), aspartate aminotransferase (AST), creatine kinase (CK), creatinine, total cholesterol (TC), low-density lipoprotein cholesterol (LDL-C), and the proportion of thrombus aspiration during PCI.

**Figure 1:**
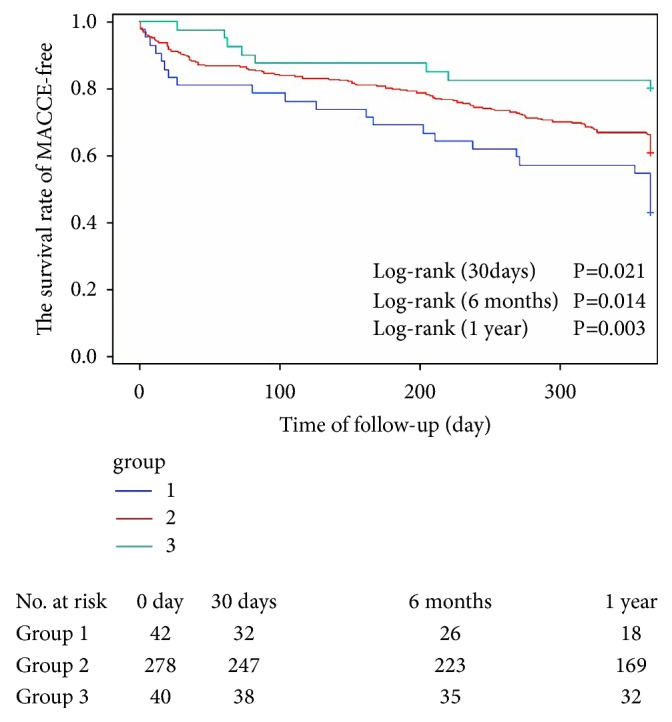
*The follow-up without a MACCE (MACCE-free) survival curve of 3 groups*. There was a statistically significant difference between the 3 groups at 30 days (0.021), 6 months (0.014), and 1 year (0.003) of follow-up. The incidence of MACCE (30 days, 6 months, and 1 year) in 3 groups increased with the decrease in the haemoglobin level.

The CAMI-STEMI scores and postoperative follow-up for each group is shown in Table 3. The Kaplan-Meier curve that depicts the follow-up without a MACCE (MACCE-free) survival curve of 3 groups is given in Figure 1. There was a statistically significant difference among the 3 groups at 30 days (0.021), 6 months (0.014) and 1 year (0.003) of follow-up. Multivariate regression analysis (Table 4) showed that the CAMI-STEMI score was an independent predictor of MACCE incidence at 30 days after PCI (HR:1.225; 95%CI:1.067-1.406; P=0.004). The Cox regression model at 30 days included the CAMI-STEMI score, haemoglobin level, albumin, thrombus aspiration during PCI, and left ventricular ejection fraction (LVEF). As is presented in Table 5, anaemia was an independent predictor of MACCE incidence at 6 months after PCI (HR:2.071; 95%CI:1.178-3.461; P=0.011). The incidence of MACCE in patients with diabetes mellitus (DM) at 6 months after PCI was higher (HR:1.709; 95%CI:1.059-2.755; P=0.028). The Cox regression model of 6 months included CAMI-STEMI score, haemoglobin level, albumin, DM, and creatinine. Multivariate regression analysis (Table 6) showed that anaemia was an independent predictor of MACCE incidence at 1 year after PCI (HR:1.521; 95%CI:0.963-2.397; P=0.071). A high haemoglobin level was an independent predictor of MACCE incidence at 1 year after PCI (HR:0.456; 95%CI:0.222-0.937; P=0.033). The incidence of MACCE in patients with DM at 1 year after PCI was higher (HR:1.420; 95%CI:0.980-2.058; P=0.063). The incidence of MACCE in patients with hypertension (HTN) at 1 year after PCI was likewise higher (HR:1.453; 95%CI:1.007-2.096; P=0.046). The incidence of MACCE in patients with thrombus aspiration during PCI at 1 year after PCI was lower (HR:0.685; 95%CI:0.489-0.961; P=0.028). The Cox regression model at 1 year included the CAMI-STEMI score, haemoglobin level, DM, HTN, albumin, history of smoking, and thrombus aspiration during PCI. The AUC of each factor for predicting the occurrence of MACCE in STEMI patients after PCI is given in Table 7.

## 4. Discussion

Anaemia is a frequently encountered comorbidity among patients presenting with ACS [14]. Liu et al showed that the risks of short-term mortality, long-term mortality, heart failure, cardiogenic shock, and major bleeding were increased in patients with anaemia compared with patients without anaemia. Anaemia may become a promising risk stratification factor in ACS [15]. Furthermore, a low baseline haemoglobin level was found to be an independent predictor of the risks of in-hospital major bleeding and of death at 1 month in ACS patients [16]. STEMI poses a serious threat to human life. Previous studies of the effect of the haemoglobin level on the prognosis of patients with coronary heart disease have mostly concentrated in ACS patients, but few studies have been conducted for STEMI patients in particular. Anaemia and high haemoglobin levels are common in STEMI patients, but the effect of the haemoglobin level on the prognosis of STEMI patients remains in dispute [1, 2]. Wang et al. reported that a haemoglobin level<120 g/L at baseline was associated with adverse outcomes and an elevated incidence of major adverse cardiovascular events (MACE) during the follow-up period in STEMI patients undergoing primary PCI [17]. Previous studies have demonstrated that the relationship between haemoglobin level and prognosis in patients with coronary heart disease was J-shaped or U-shaped, which indicated that patients with a high haemoglobin level have poor prognosis [18].

This study showed that the levels of haemoglobin in male and smoking STEMI patients were higher and that the concentration of haemoglobin decreased with age. Albumin, ALT, AST, CK, TC, LDL-C, and the proportion of thrombus aspiration during PCI were lower in STEMI patients with low levels of haemoglobin; and creatinine and the proportion of Killip class greater than 1 were higher in STEMI patients with high levels of haemoglobin. The Kaplan-Meier curve (Figure 1) showed that the incidence of MACCE in the 3 groups increased with the decrease of haemoglobin level within 30 days, 6 months, and 1 year after PCI. The CAMI-STEMI score is a newly published, simple, and practical risk stratification scoring system. It does not require blood tests and medical history. Predictive accuracy of hospitalized mortality in Chinese STEMI patients is similar to that obtained using TIMI score and the Global Registration of Acute Coronary Events (GRACE) score [10]. This study showed that the CAMI-STEMI score was an independent predictor of MACCE incidence at 30 days after PCI. Haemoglobin was the most widely used experimental datum in STEMI patients, and the result was stable and easy to observe. This study showed that anaemia was an independent predictor of MACCE incidence at 6 months and 1 year after PCI and that the risk of MACCE incidence at 6 months and 1 year after PCI was significantly higher than that of patients with normal haemoglobin levels. The probable reasons for the worse clinical outcome and mortality in anaemic patients with AMI are as follows. Anaemia leads to the reduction of the myocardial oxygen supply and the increased myocardial oxygen demand, which have a negative effect on the myocardium [19]. Anaemia also leads to volume expansion, thereby contributing to the development of chronic heart failure (CHF) [20]. High haemoglobin levels were shown to increase blood viscosity, which, in turn, caused increased coronary vascular resistance, decreased coronary blood flow, and a predisposition to thrombosis [21–23]. However, this study showed that a high haemoglobin level was an independent predictor of MACCE incidence at 1 year after PCI (HR:0.456; 95%CI:0.222-0.937; P=0.033) and was a protective factor. This may be linked to the fact that increases in haemoglobin could elevate systemic vascular resistance and increase blood pressure [24, 25]. Gevaert et al. [26] showed that a systolic blood pressure less than 100 mmHg increased mortality by 3.5 times among STEMI patients. Meanwhile, high haemoglobin carried more oxygen and increased oxygen delivery to myocardium downstream of coronary stenoses. This study also indicated that the haemoglobin level and CAMI-STEMI score had a high predictive value for MACCE in STEMI patients within 30 days after PCI and that the haemoglobin level combined with the CAMI-STEMI score improved the predictive value of MACCE in STEMI patients within 30 days after PCI. By contrast, CAMI-STEMI score had a poor predictive value for MACCE in STEMI patients at 1 year, whereas the predictive value of the CAMI-STEMI score declined with time.

## 5. Study Limitations

The following limitations of the present study should be addressed. First, the number of patients included in this study was relatively limited. There was a selection bias, and whether these defects will influence the results also requires a prospective study of large samples. Second, in this study, we only included the hemoglobin 24 hours after PCI and the etiology of anaemia or low values of haemoglobin and the detail of procedural complications were not reported. Third, although we adjusted several known confounding variables in the multivariable Cox proportional hazards models, other unknown factors might have played roles in MACCE.

## 6. Conclusion

This study indicated that the incidence of MACCE increased with a decrease in the haemoglobin level in STEMI patients. Anaemia was an independent predictor of MACCE incidence at 6 months and 1 year after PCI. And a high haemoglobin level was an independent predictor of MACCE incidence at 1 year after PCI and was a protective factor. The CAMI-STEMI score was an independent predictor of MACCE incidence at 30 days after PCI. The haemoglobin level combined with the CAMI-STEMI score improved the predictive value of MACCE in STEMI patients within 30 days after PCI. The American Heart Association (ACC) and American College of Cardiology (AHA) guidelines recommend screening and correcting anaemia in ACS patients [27, 28]. Owing to the lack of adequate clinical data support, the guidelines did not specify which haemoglobin level to target. Greater sample size, more haemoglobin subgroups, longer follow-up time, and multicentre trials are needed to further determine the ideal haemoglobin level for STEMI patients and to provide ideas for optimizing the treatment of myocardial infarction and improving the prognosis of patients.

## Figures and Tables

**Table 1:** Baseline clinical data.

Variable	Group1 (n=42)	Group2 (n=278)	Group3 (n=40)	P Value
Female sex	17 (40.5%)	65 (23.4%)	1 (2.5%)	<0.001
Age, year	71.7±9.9	63.3±12.6	51.1±9.8	<0.001
Heart rate, bpm	81.3±17.3	78.8±15.4	83.2±12.4	0.148
Systolic blood pressure, mmHg	118.6±23.0	130.9±56.1	140.7±22.2	0.139
Diastolic blood pressure, mmHg	86.1±16.9	82.9±21.5	87.1±17.9	0.888
Killip class>1	18 (12.9%)	50 (18.0%)	2 (5%)	<0.001
Sudden cardiac arrest	1 (2.4%)	1 (0.4%)	0 (0%)	0.142
Anterior myocardial infarction	18 (42.9%)	127 (45.7%)	25 (62.5%)	0.115
Body mass index, kg/m^2^	22.91±3.36	24.51±3.37	25.65±3.07	0.010
Smoker	28 (66.7%)	152 (54.7%)	12 (30%)	0.003
Hypertension	27 (64.3%)	171 (61.5%)	27 (67.5)	0.834
Diabetes mellitus	16 (38.1%)	66 (23.7%)	9 (22.5%)	0.125

Data are presented as the mean±SD or n (%).

**Table 2:** Biochemical and angiographic variables and ultrasonic cardiogram results.

Variable	Group 1 (n=42)	Group 2 (n=278)	Group 3 (n=40)	P Value
Biochemical indicators				
Haemoglobin, g/L (IQR)	108(99-112)	139(130-147)	168(161-174)	<0.001
Albumin, g/L	32.5±5.3	37.4±4.5	39.4±5.4	<0.001
ALT, IU/L (IQR)	26(15-53)	37(25-58)	48(34-82)	0.001
AST, IU/L (IQR)	85(31-141)	112(46-202)	114(59-266)	0.034
CK, IU/L (IQR)	560(139-1288)	1028(279-2043)	1249(275-2267)	0.018
Cardiac troponin I, ng/ml (IQR)	5.13(3.22-6.84)	3.85(1.13-6.46)	4.19(2.31-7.09)	0.276
CK-MB, ng/ml (IQR)	82(29.6-303.3)	83(32.5-318.2)	82(26.8-310.9)	0.539
Glucose, mmol/L (IQR)	7.3(5.7-10.8)	7.5(6.4-9.4)	7.8(6.6-11.0)	0.355
Glycosylated haemoglobin, %	5.7±2.0	5.9±1.7	6.0±1.3	0.768
Creatinine, mmol/L (IQR)	107(72-141.5)	81(69-97)	85.5(69.5-100)	0.006
eGFR, mL/min	73.1±30.2	75.2±33.4	81.3±30.9	0.201
TC, mmol/L (IQR)	3.7(3.4-4.4)	4.6(3.9-5.4)	4.8(4.3-5.6)	<0.001
LDL-C, mmol/L (IQR)	2.4(2.0-2.7)	2.9(2.4-3.4)	2.8(2.5-3.4)	0.001
Uric acid, mmol/L (IQR)	342(269-404)	310(252-377)	344(287-393)	0.056
Coronary angiography				
Successful PCI	41(97.6%)	271(97.5%)	39(97.5%)	0.999
Door-to-balloon time, min	51.5±22.4	50.0±17.9	48.4±25.8	0.447
Trans-radial	35(83.3%)	264(95.0%)	39(97.5%)	0.039
Three-vessel disease	20(47.6%)	146(52.5%)	13(32.5%)	0.058
Coronary thrombi	19(45.2%)	169(60.8%)	27(67.5%)	0.091
Thrombus aspiration	16(38.1%)	162(58.3%)	26(65.0%)	0.026
Ultrasonic cardiogram				
LVEF (IQR)	0.53(0.4-0.62)	0.54(0.47-0.61)	0.56(0.5-0.63)	0.242
Regional wall motion abnormality	27(64.3%)	200(71.9%)	30(75%)	0.513
Medication use at discharge				
Aspirin	39(92.9%)	272(97.8%)	38(95.0%)	0.453
Clopidogrel/ Ticagrelor	42(100%)	274(98.6%)	40(100%)	0.551
Statin	40(95.2%)	269(96.8%)	39(97.5%)	0.293
Beta blockers	32(76.2%)	221(79.5%)	33(82.5%)	0.311
ACEI/ARB	20(47.6%)	182(65.5%)	28(70.0%)	0.062

*Abbreviations.* IQR = interquartile range; ALT = alanine aminotransferase; AST = aspartate aminotransferase; CK = creatine kinase; CK-MB = creatinine kinase-MB; eGFR = estimated glomerular filtration rate; TC = total cholesterol; LDL-C = low-density lipoprotein cholesterol; PCI = percutaneous coronary intervention; LVEF = left ventricular ejection fraction. ACEI=angiotensin converting enzyme inhibitor; ARB= angiotensin receptor blocker. Data are presented as the mean±SD, median (IQR), or n (%).

**Table 3:** CAMI-STEMI score and follow-up results.

Variable	Group 1 (n=42)	Group 2 (n=278)	Group 3 (n=40)	P Value
CAMI-STEMI score (IQR)	4(3-6)	2(1-4)	1(0.5-1)	<0.001*∗*
MACCE occurrence (30 days after PCI)	10(23.8%)	31(11.2%)	2(5.0%)	0.021*∗∗*
MACCE occurrence (6 months after PCI)	16(38.1%)	57(20.5%)	5(12.5%)	0.014*∗∗*
MACCE occurrence (1 year after PCI)	24(57.1%)	109(39.2%)	8(20.0%)	0.003*∗∗*

*Abbreviations.* IQR = interquartile range. Data are presented as median (IQR) or n (%).

*∗*There was a statistically significant difference in CAMI-STEMI score between the 3 groups.

*∗∗*There was a statistically significant difference in MACCE occurrence between the 3 groups at 30 days (0.021), 6 months (0.014), and 1 year (0.003) of follow-up. The incidence of MACCE (30 days, 6 months, and 1 year) in the 3 groups increased with the decrease in the haemoglobin level.

**Table 4:** Univariate and multivariate analysis and predictors for 30-day MACCE.

Variable	Univariate analysis			Multivariate analysis		
	HR	95%CI	P Value	HR	95%CI	P Value
Haemoglobin grouping						
Group 2	1					
Group 1	2.258	1.107- 4.607	0.025	3.515	0.643-19.216	0.147
Group 3	0.427	0.102- 1.785	0.244	0.549	0.124-2.498	0.438
CAMI-STEMI score	1.203	1.060-1.365	0.004	1.225	1.067- 1.406	0.004*∗*
Body mass index	1.103	0.812-1.394	0.552			
Diabetes mellitus	1.319	0.688- 2.525	0.405			
Smoker	1.542	0.831- 2.861	0.170			
Albumin	0.915	0.865- 0.969	0.002	0.997	0.990-1.003	0.823
Creatinine	1.002	1.000- 1.005	0.074	1.003	0.992-1.011	0.783
TC	0.765	0.569- 1.029	0.077	1.000	0.999-1.002	0.609
LDL-C	0.737	0.484- 1.122	0.155			
Uric acid	1.002	0.999- 1.005	0.136			
Glucose	1.042	0.984-1.104	0.159			
Thrombus aspiration	0.524	0.286- 0.962	0.037	0.564	0.289-1.101	0.094
Trans-radial	0.975	0.824-1.121	0.738			
LVEF	0.270	0.001-0.521	0.017	0.795	0.134-1.225	0.211

*Abbreviations.* TC = total cholesterol; LDL-C = low-density lipoprotein cholesterol; LVEF = left ventricular ejection fraction.

*∗*The CAMI-STEMI score was an independent predictor of MACCE incidence at 30 days after PCI (HR:1.225; 95%CI:1.067-1.406; P=0.004).

**Table 5:** Univariate and multivariate analyses and predictors for 6-month MACCE.

Variable	Univariate analysis			Multivariate analysis		
	HR	95%CI	P Value	HR	95%CI	P Value
Haemoglobin grouping						
Group 2	1					
Group 1	1.978	1.136-3.444	0.016	2.071	1.178-3.461	0.011**∗**
Group 3	0.574	0.230-1.431	0.233	0.691	0.250-1.908	0.476
CAMI-STEMI score	1.150	1.043- 1.267	0.005	1.073	0.943-1.221	0.287
Body mass index	1.093	0.916-1.167	0.338			
Diabetes mellitus	1.767	1.112-2.809	0.016	1.709	1.059-2.755	0.028*∗∗*
Smoker	1.557	0.984-2.464	0.059	1.634	0.920-2.907	0.094
Albumin	0.922	0.883- 0.963	<0.001	0.995	0.990-1.013	0.745
Creatinine	1.002	1.000- 1.004	0.035	1.001	0.999-1.002	0.584
TC	0.855	0.692-1.058	0.150			
LDL-C	0.840	0.612- 1.153	0.281			
Uric acid	1.002	1.000-1.004	0.118			
Glucose	1.050	1.007- 1.095	0.022	1.001	0.997-1.004	0.896
Thrombus aspiration	0.657	0.422-1.026	0.065	0.870	0.416-0.195	0.194
Trans-radial	0.934	0.723-1.224	0.832			
LVEF	0.279	0.032-2.422	0.247			

*Abbreviations.* TC = total cholesterol; LDL-C = low-density lipoprotein cholesterol; LVEF = left ventricular ejection fraction.

*∗*Anaemia was an independent predictor of MACCE incidence at 6 months after PCI (HR:2.071; 95%CI:1.178-3.461; P=0.011). *∗∗*The incidence of MACCE in patients with diabetes mellitus at 6 months after PCI was higher (HR:1.709; 95%CI:1.059-2.755; P=0.028).

**Table 6:** Univariate and multivariate analyses and predictors for 1-year MACCE.

Variable	Univariate analysis			Multivariate analysis		
	HR	95%CI	P Value	HR	95%CI	P Value
Haemoglobin grouping						
Group 2	1					
Group 1	1.659	1.066-2.581	0.025	1.521	0.963-2.397	0.071*∗*
Group 3	0.455	0.222-0.934	0.032	0.456	0.222-0.937	0.033*∗∗*
CAMI-STEMI score	1.069	0.990-1.154	0.088	1.046	0.933-1.171	0.443
Body mass index	1.047	0.953-1.033	0.674			
Diabetes mellitus	1.524	1.067-2.179	0.020	1.420	0.980-2.058	0.063*∗∗∗∗*
Smoker	1.495	1.066- 2.098	0.020	1.503	0.944-2.394	0.086
Albumin	0.966	0.934-0.999	0.046	0.999	0.998-1.001	0.408
Creatinine	1.001	0.999-1.003	0.194			
TC	0.952	0.818-1.107	0.522			
LDL-C	0.860	0.687-1.077	0.188			
Uric acid	1.002	1.000-1.003	0.010	1.003	0.998-1.007	0.079
Glucose	1.020	0.983-1.058	0.298			
Thrombus aspiration	0.663	0.476-0.923	0.015	0.685	0.489-0.961	0.028*∗∗∗∗∗*
Trans-radial	0.977	0.663-1.376	0.732			
LVEF	2.400	0.499-11.540	0.275			

*Abbreviations.* TC = total cholesterol; LDL-C = low-density lipoprotein cholesterol; LVEF = left ventricular ejection fraction.

*∗*Anaemia was an independent predictor of MACCE incidence at 1 year after PCI (HR:1.521; 95%CI:0.963-2.397; P=0.071).*∗∗*A high haemoglobin level was an independent predictor of MACCE incidence at 1 year after PCI (HR:0.456; 95%CI:0.222-0.937; P=0.033). *∗∗∗*The incidence of MACCE in patients with hypertension at 1 year after PCI was also higher (HR:1.453; 95%CI:1.007-2.096; P=0.046). *∗∗∗∗*The incidence of MACCE in patients with diabetes mellitus at 1 year after PCI was higher (HR:1.420; 95%CI:0.980-2.058; P=0.063). *∗∗∗∗∗*The incidence of MACCE in patients with thrombus aspiration during PCI at 1 year after PCI was lower (HR:0.685; 95%CI:0.489-0.961; P=0.028).

**Table 7:** The AUC of each factor predicting the occurrence of MACCE in STEMI patients after PCI.

Variable	30 days		6 months		1 year	
	AUC	95%CI	AUC	95%CI	AUC	95%CI
CAMI-STEMI score	0.614	0.587-0.644	0.585	0.564-0.605	0.538	0.524-0.554
Haemoglobin level	0.604	0.574-0.632	0.589	0.566-0.611	0.578	0.563-0.592
Haemoglobin level combined with CAMI-STEMI score	0.639	0.610-0.667	0.609	0.587-0.629	0.582	0.567-0.596

*Abbreviations.* AUC = the area under receiver operating characteristic curve; MACCE = the main adverse cardiac and cerebrovascular events; STEMI = ST Elevation Myocardial Infarction; PCI = percutaneous coronary intervention; CAMI-STEMI score = the China Acute Myocardial Infarction registry-ST Elevation Myocardial Infarction score.

It showed that the haemoglobin level and CAMI-STEMI score had a high predictive value for MACCE in STEMI patients within 30 days after PCI (AUC 0.604, 0.614) and that the haemoglobin level combined with CAMI-STEMI score could improve the predictive value of MACCE in STEMI patients within 30 days after PCI (AUC 0.639). In contrast, CAMI-STEMI score had a poor predictive value for MACCE in STEMI patients at 1 year (AUC 0.538).

## Data Availability

The datasets used and/or analyzed during the present study are available from the corresponding author on reasonable request.
